# Primary Care Physicians' Knowledge and Perceptions of Bariatric Surgery: A Cross-Sectional Analysis

**DOI:** 10.7759/cureus.90719

**Published:** 2025-08-22

**Authors:** Qin Sun, Junming Cheng, Yuan Zhang, Ming He, Jiebin Xie

**Affiliations:** 1 Discipline Inspection Commission Office, North Sichuan Medical College, Nanchong, CHN; 2 Department of Gastroenterology, Dazhu County People's Hospital, Dazhu, CHN; 3 Department of Gastroenterology, Affiliated Hospital of North Sichuan Medical College, Nanchong, CHN; 4 Department of Gastrointestinal Surgery, Affiliated Hospital of North Sichuan Medical College, Nanchong, CHN

**Keywords:** bariatric surgery, knowledge, obesity, primary care doctors, public health

## Abstract

Objective

The purpose of this study is to assess the awareness of primary care doctors in China about obesity and bariatric surgery.

Methods

Between September and December 2024, an anonymous electronic questionnaire was distributed to doctors in primary care hospitals. The questionnaire included four sections: basic information, knowledge about obesity, understanding of weight loss surgery, and factors affecting the implementation of bariatric surgery, comprising a total of 34 questions. After the surveys were collected, data were compiled and analyzed according to participants’ gender, age, education, title, hospital, and department.

Results

Among the respondents, 50.3% were male and 49.7% were female, with the majority being aged 31-40 years, and most holding a bachelor's degree. About 79.4% of respondents had not received training in bariatric surgery, and 85.2% had not participated in postoperative follow-up. About 54.8% of hospitals did not offer bariatric surgery, and 71.6% did not provide related educational activities. Most respondents believed obesity is a chronic disease related to diabetes and cardiovascular diseases, and recognized that obesity is associated with economic status and family social standing. Regarding bariatric surgery, 25.2% of respondents considered it the best treatment option, 40.7% recommended that family members consider the surgery, and 43.2% believed long-term management is necessary after the surgery.

Conclusion

Primary care doctors in community hospitals have inadequate awareness of obesity and bariatric surgery, and most community hospitals do not prioritize obesity enough.

## Introduction

The World Obesity Atlas 2024, published by the World Obesity Federation (WOF), projects that by 2035, 3.3 billion adults - equivalent to 54% of the global adult population - will be affected by overweight and obesity [1]. According to the China Obesity Prevention and Treatment and Bariatric Surgery White Paper 2023, the average BMI and waist circumference of Chinese adults continue to increase, with the combined overweight and obesity rate exceeding 50%. Based on Chinese diagnostic criteria (BMI ≥ 28 kg/m²), China has the largest number of obese individuals globally. The obesity rate in China continues to rise, posing a serious threat to public health and constituting an urgent public health issue [2, 3]. Obesity affects nearly all organ systems and is associated with an increased risk of chronic diseases, disability, and premature mortality [4-7]. Bariatric surgery, an effective and durable intervention for obesity and related metabolic disorders, has gained broad clinical acceptance and is now widely implemented in practice [8]. Current research primarily focuses on patient acceptance and the long-term outcomes of bariatric surgery. However, the extent of clinicians’ understanding of obesity and bariatric surgery - despite their central role in healthcare decision-making - remains unclear. This study investigates Chinese primary care physicians’ awareness of bariatric surgery, aiming to improve medical guidance for obese patients and strengthen the scientific rigor and accuracy of clinical decision-making.

## Materials and methods

This study distributed anonymous electronic questionnaires to primary care physicians in community hospitals between September and December 2024, and used a self-designed questionnaire, which was structured based on the White Paper on Obesity Prevention and Bariatric Surgery in China (2023) and the WHO Obesity Management Guidelines (see Supplementary Material in the Appendices). This study surveyed general practitioners from community hospitals in Nanchong using an anonymous electronic questionnaire. Data were collected between 1 September 2024 and 31 December 2024, spanning four months and covering community hospitals across multiple provinces. After removing incomplete responses, 155 valid questionnaires were collected. The survey consisted of four sections with a total of 34 questions. The first section, "Basic Information," contained 13 questions, including gender, age, education, title, region, hospital level, department, whether the participant had received bariatric surgery training, and related details. The second section, "Knowledge of Obesity," included nine questions related to obesity and its associated conditions. The third section, "Knowledge of Bariatric Surgery," contained 11 questions focusing on professional knowledge related to weight loss surgery. The fourth section, "Factors Affecting Bariatric Surgery," consisted of four questions. After the questionnaires were collected, data were compiled and analyzed according to participants' gender, age, education, title, hospital, and department. Participants were classified into four age groups: 20-30, 31-40, 41-50, and 51-60 years. Education level groups included associate degree, bachelor's degree, master's degree, and doctorate. Job titles were grouped into four levels: resident physician, attending physician, associate chief physician, and chief physician. Hospitals were categorized into three groups: private hospitals, secondary A and below hospitals, tertiary B hospitals, and tertiary A hospitals.

Inclusion criteria: 1. Participants were general practitioners in Chinese community hospitals, 2. Participants were willing to participate and signed the electronic informed consent form.

Exclusion criteria: 1. Incomplete questionnaires (missing rate > 10%), 2. Doctors in non-clinical departments or not directly involved in patient management.

Stratified random sampling was used, and questionnaire links were assigned according to hospital level and region. Questionnaires were distributed via WeChat and email through the department heads of each community hospital to their general practitioners. Invitations were sent between 09:00 and 18:00 on working days, and participants could complete the survey on their personal devices in up to 15 minutes.

This study was approved by the Ethics Committee of the Affiliated Hospital of North Sichuan Medical College (2025ER78-1).

Statistical analysis

All analyses were performed with SPSS 23.0 (IBM Corp., Armonk, NY, USA). Since every variable in this study was categorical (binary or polytomous), between-group comparisons were carried out exclusively with χ² tests; whenever an expected cell frequency was <5, Fisher’s exact test was used instead. Results are presented as counts (n) and percentages (%). Two-sided P-values <0.05 were considered statistically significant. Figures were generated in R 4.3.0 (R Foundation for Statistical Computing, Vienna, Austria) using the ggplot2, tidyr, and dplyr packages to produce stacked bar charts.

## Results

Basic information

A total of 155 physicians participated in this study, including 78 males (50.3%) and 77 females (49.7%). Most participants were aged 31-40 years (n = 76, 49.1%). The majority held a bachelor’s degree (n = 82, 55.1%), followed by a master’s degree (n = 60, 40.3%). Attending physicians accounted for the largest proportion of participants (n = 63, 44.3%). Most respondents were employed at tertiary grade B hospitals (n = 93, 63.3%) (Table 1).

**Table 1:** Providers’ Demographics

Variables	Numbers (%)	Test statistic	P value
Gender		χ² = 0.006	0.936
Male	78 (50.3%)		
Female	77 (49.7%)		
Age		χ² = 92.3	＜0.001
20-30	19 (12.2%)		
31-40	76 (49.1%)		
41-50	38 (24.5%)		
51-60	22 (14.2%)		
Educational Attainment		χ² = 138.5	< 0.001
Associate degree	2 (1.3%)		
Bachelor's degree	82 (55.1%)		
Master's degree	60 (40.3%)		
Doctoral degree / Doctor of Philosophy	5 (3.3%)		
Medical Professional Title		χ² = 47.84	< 0.001
Resident physician	18 (12.7%)		
Attending physician	63 (44.3%)		
Associate chief physician	49 (34.5%)		
Chief physician	12 (8.5%)		
Hospital Grade of Providers		χ² = 120.26	< 0.001
Second-class grade B hospital	2 (1.3%)		
Second-class grade A hospital	29 (19.7%)		
Third-class grade B hospital	93 (63.3%)		
Third-class grade A hospital	23 (15.7%)		

Univariate analysis showed no significant association between gender and awareness or recommendation of bariatric surgery (χ² = 0.006, p = 0.936). However, age, educational attainment, professional title, and hospital grade were significantly associated with bariatric surgery-related knowledge (p < 0.001). The table highlights several key findings: the largest proportion of participants is aged 31-40 years (49.1%), with a significant difference (χ² = 92.3, P < 0.001). In terms of medical professional title, attending physicians make up the highest percentage (44.3%) with a significant difference (χ² = 47.84, P < 0.001), and most participants work in third-class grade B hospitals (63.3%) with a significant difference (χ² = 120.26, P < 0.001). These categories show the most notable distributions in the study.

Participation in bariatric surgery training and follow-up monitoring

The results showed that 123 participants (79.4%) had not received any training in bariatric surgery, and 132 (85.2%) had never participated in postoperative follow-up for bariatric procedures (Table 2).

**Table 2:** Involvement in Bariatric Surgery Training and Follow-up Activities χ² tests assumed H₀: equal distribution across categories

Questions	Numbers (%)		Test statistic	P value
	Yes	No		
Whether you have received bariatric surgery training	32 (20.6%)	123 (79.4%)	χ² = 106.8	< 0.001
Whether you have participated in bariatric surgery follow-up	23 (14.8%)	132 (85.2%)	χ² = 142.2	< 0.001

Univariate analysis indicated that both variables were significantly associated with physician knowledge and attitudes toward bariatric surgery (training: χ² = 106.8, p < 0.001; follow-up: χ² = 142.2, p < 0.001). Physicians who had received training were significantly more likely to recommend bariatric surgery to eligible patients, and those who had participated in follow-up were more likely to recognize the need for long-term postoperative management.

Bariatric surgery and related educational initiatives at the hospital

Among the surveyed physicians, 85 (54.8%) reported that bariatric surgery was not available in their hospitals, and 111 (71.6%) stated that no educational activities related to bariatric surgery were provided to patients. Only 35 participants (22.6%) indicated that their institutions organized educational events on obesity and bariatric surgery, and just 37 (23.9%) confirmed that their hospitals conducted awareness campaigns on these topics (Table 3).

**Table 3:** Availability of Bariatric Surgery and Related Educational Activities in Hospitals χ² tests assumed H₀: equal distribution across categories

Questions	Numbers (%)		Test statistic	P value
	Yes	No		
Whether there is bariatric surgery available in the hospital where one is located	70 (45.2%)	85 (54.8%)	χ² = 2.1	0.228
Whether there are educational activities for patients related to bariatric surgery in the hospital where one is located	44 (28.4%)	111 (71.6%)	χ² = 45.3	< 0.001
Whether the hospital where one is located holds education related to obesity and bariatric surgery	35 (22.6%)	120 (77.4%)	χ² = 75.6	< 0.001
The hospital and relevant departments will conduct publicity on obesity and bariatric surgery.	37 (23.9%)	118 (76.1%)	χ² = 70.2	< 0.001

Univariate analyses revealed that institutional educational support was significantly associated with physician knowledge and proactive attitudes toward bariatric surgery. Specifically, the presence of patient education programs on bariatric surgery was linked to a greater likelihood of recommending surgery (χ² = 45.3, p < 0.001). Similarly, hospitals that conducted educational events and campaigns on obesity and bariatric surgery had physicians with significantly higher awareness scores (education events: χ² = 75.6, p < 0.001; awareness campaigns: χ² = 70.2, p < 0.001).

No significant association was found between the availability of bariatric surgery in the hospital and physicians’ recommendation behavior (χ² = 2.1, p = 0.228).

Results on the influence of gender, age, education level, and hospital background on knowledge of obesity and bariatric surgery

The findings revealed significant disparities in participants' understanding of obesity and its associated factors. Regarding perceptions of obesity, the vast majority of participants (n=92, 59.4%) recognized obesity as a chronic disease, with 104 (67.1%) agreeing that obesity exacerbates diabetes and cardiovascular diseases. Most respondents believed obesity was related to household economic status (n=64, 41.3%) and local economic conditions (n=65, 41.9%). Additionally, approximately 60 (38.8%) of participants associated obesity with family social status, while about 41.9% perceived a connection between parental education levels and childhood obesity. In terms of lifestyle modifications, 61 (39.4%) of participants recommended prioritizing lifestyle changes as the primary approach to address obesity (Table 4).

**Table 4:** Views on Obesity

Questions	Numbers (%)				
	Strongly agree	Agree	Neutral	Disagree	Strongly disagree
Obesity is a chronic disease	3 (1.9%)	92 (59.4%)	40 (25.8%)	16 (10.3%)	4 (2.6%)
There is a relationship between obesity and family economic level	29 (18.7%)	64 (41.3%)	31 (20.0%)	30 (19.4%)	1 (0.6%)
There is a relationship between obesity and local economic level	31 (20.0%)	65 (41.9%)	33 (21.3%)	26 (16.8%)	0 (0.0%)
There is a relationship between family social status and obesity	32 (20.6%)	60 (38.8%)	32 (20.6%)	31 (20.0%)	0 (0.0%)
There is a relationship between parents' educational attainment and childhood obesity	38 (24.5%)	78 (50.4%)	35 (22.6%)	3 (1.9%)	1 (0.6%)
Obesity exacerbates diabetes and cardiovascular diseases	104 (67.1%)	32 (20.6%)	14 (9.1%)	5 (3.2%)	0 (0.0%)
Recommend changing the lifestyle as the top priority	61 (39.4%)	59 (38.1%)	19 (12.2%)	9 (5.8%)	7 (4.5%)
Patients with obesity are more concerned about the diseases that are easiest to solve and most directly related to their goals.	76 (49.0%)	37 (23.9%)	21 (13.5%)	11 (7.1%)	10 (6.5%)

**Figure 1:**
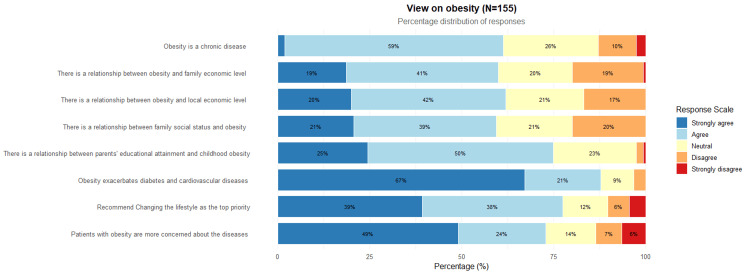
Views on Obesity

Univariate analysis showed that physicians with public health training were significantly more likely to agree that obesity is a chronic disease. Similarly, younger physicians (age 31-40) were more inclined to associate obesity with socioeconomic factors such as family income and parental education level (Figure 1).

In addition, 39 (25.2%) of participants believed that weight loss surgery is the best option for treating obesity, and 62 (40.0%) thought it could cure metabolic diseases caused by obesity; 58 (37.4%) of participants considered weight loss surgery as the last treatment option for obese patients, while 59 (38.1%) thought the surgery was suitable for those with severe obesity. Regarding the promotion of weight loss surgery, 63 (40.7%) of participants recommended that family members of obese patients consider the surgery, but only 39 (25.2%) would proactively recommend weight loss surgery to patients with obesity-related complications. Regarding long-term management after surgery, 67 (43.2%) of participants believed that long-term management is needed after weight loss surgery, and 38 (24.5%) thought lifelong supplementation of vitamins and trace elements is necessary post-surgery (Table 5).

**Table 5:** Views on Bariatric Surgery

Questions	Numbers (%)				
	Strongly agree	Agree	Neutral	Disagree	Strongly disagree
Bariatric surgery is the best option for the treatment of obesity	12 (7.8%)	39 (25.2%)	79 (50.9%)	24 (15.5%)	1 (0.6%)
Bariatric surgery can cure metabolic diseases caused by obesity	14 (9.1%)	65 (41.9%)	46 (29.6%)	29 (18.8%)	1 (0.6%)
Bariatric surgery is a neurohumoral intervention	2 (1.2%)	62 (40.0%)	61 (39.4%)	30 (19.4%)	0 (0.0%)
Bariatric surgery is the last treatment option for patients with obesity	58 (37.4%)	30 (19.4%)	50 (32.3%)	15 (9.7%)	2 (1.2%)
Bariatric surgery is beneficial for the treatment of obesity-related comorbidities	59 (38.1%)	23 (14.8%)	64 (41.3%)	9 (5.8%)	0 (0.0%)
Actively recommend bariatric surgery for patients with obesity and comorbidities	39 (25.2%)	39 (25.2%)	65 (41.9)	11 (7.1%)	1 (0.6%)
Recommend bariatric surgery to family members of patients with obesity genes	63 (40.7%)	2 (1.2%)	84 (54.2%)	6 (3.9%)	0 (0.0%)
Be aware of the indications for bariatric surgery	19 (12.3%)	30 (19.4%)	16 (10.3%)	52 (33.5%)	38 (24.5%)
Be aware of many side effects of bariatric surgery	26 (16.8%)	38 (24.5%)	37 (23.9%)	29 (18.7%)	25 (16.1%)
Long-term management is required after bariatric surgery	67 (43.2%)	42 (27.1%)	39 (25.2%)	6 (3.9%)	1 (0.6%)
After bariatric surgery, vitamins and trace elements need to be taken throughout one's life	38 (24.5%)	41 (26.5%)	62 (40.0%)	9 (5.8%)	5 (3.2%)
Have an understanding of endoscopic weight loss and metabolic therapy	12 (7.8%)	31 (20.0%)	44 (28.4%)	36 (23.2%)	32 (20.6%)
Take the initiative to learn about the cutting-edge knowledge of obesity and bariatric surgery	48 (30.9%)	68 (43.9%)	28 (18.2%)	9 (5.8%)	2 (1.2%)
Recommend obese patients to learn about bariatric surgery	37 (23.9%)	32 (20.7%)	63 (40.6%)	18 (11.6%)	5 (3.2%)
Bariatric surgery is suitable for all severely obese patients	23 (14.8%)	41 (26.5%)	36 (23.2%)	28 (18.1%)	27 (17.4%)

In terms of long-term management, 70.3% of participants recognized the need for ongoing follow-up after bariatric surgery, while only 24.5% strongly agreed that lifelong supplementation with vitamins and trace elements is necessary. Additionally, a substantial proportion of participants reported insufficient understanding of endoscopic metabolic therapies (43.8% disagree or strongly disagree), highlighting a gap in knowledge regarding newer approaches.

**Figure 2:**
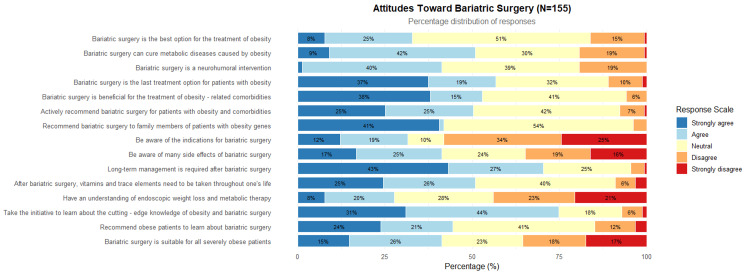
Views on Bariatric Surgery

Physicians who had undergone bariatric training were significantly more likely to: Actively recommend surgery to patients with comorbidities; be aware of surgical indications; support long-term nutritional management. These findings suggest that professional training plays a critical role in shaping physician attitudes and behaviors toward bariatric surgery (Figure 2).

## Discussion

Obesity has been designated by the World Health Organization as “one of the most serious chronic non-communicable diseases of the 21st century.” Data from the Global Burden of Disease Study show that over the past three decades, the prevalence of obesity has nearly tripled, and by 2030, approximately one-third of the world’s adults will meet obesity criteria [9-10]. In China, the 2023 White Paper on Obesity Prevention and Treatment & Bariatric Surgery reports that the combined prevalence of overweight and obesity among adults has already exceeded 50% and is continuing to rise by 1.2-1.5 percentage points annually [2,3]. This epidemiological trend not only drives up the incidence of type 2 diabetes mellitus, non-alcoholic fatty liver disease, hypertension, polycystic ovary syndrome, and at least 13 malignancies, but also causes direct medical expenditures and indirect productivity losses to grow exponentially [11-15]. Confronted with a health and economic crisis of this magnitude, bariatric surgery (BS) - the only intervention proven to achieve sustained weight loss and markedly improve metabolic comorbidities in patients with severe obesity - is used in less than 1% of eligible individuals worldwide, creating a vast “evidence-to-practice gap” [16,17].

In the United States, for example, although the Centers for Medicare & Medicaid Services (CMS) expanded coverage for BS in 2023 and more than 900 accredited centers now perform the procedure, 43.9% of respondents in national surveys still believe that “surgical risks outweigh benefits” [18,19]. The situation in Europe is similarly sobering: the 2022 report from Sweden’s National Board of Health and Welfare indicates that, despite a well-developed primary-care system and a national bariatric registry, only 28% of primary-care physicians are willing to refer eligible patients to bariatric centers, with regional variation as high as 3.4-fold [20-25]. When data from Asia, Latin America, and resource-limited African countries are examined, surgical rates remain below 0.3%, with the principal barriers being lack of trained teams, inadequate patient purchasing power, cultural stigma, and distrust in long-term follow-up systems [26-30]. Thus, low utilization of BS is not attributable to a single country or factor, but rather results from the complex interaction of cultural norms, educational deficits, health policies, economic incentives, and information systems [31,32].

At the provider level, multicenter studies consistently identify a broken “knowledge-attitude-practice” chain as the foremost bottleneck: (1) insufficient mastery of surgical indications and contraindications - globally, only 35-40% of primary-care physicians can accurately state BMI thresholds and comorbidity criteria; (2) lack of confidence in peri-operative risk assessment and complication management - about 60% of physicians overestimate early complication rates by two- to three-fold; and (3) poor appreciation of long-term follow-up and nutritional management - only 20-30% of physicians are aware that lifelong micronutrient supplementation is required [23]. These deficiencies directly depress referral willingness and perpetuate a vicious cycle in which “physicians do not recommend → patients remain uninformed → surgical volumes stay low → training opportunities shrink.”

To address these challenges, over the past decade, the Endocrine Society, the European Association for the Study of Obesity, and the International Federation for the Surgery of Obesity and Metabolic Disorders (IFSO) have launched structured continuing medical education (CME) programs. Using online case simulations, multidisciplinary workshops, and rotations in accredited bariatric centers, these initiatives have increased physician referral rates for BS by 1.8-2.5-fold [33-36]. In parallel, Sweden has embedded “obesity navigators” in primary-care clinics - nurses or dietitians who receive 40 hours of CME and are tasked with screening and patient education - boosting the referral rate to 54% [24]. In resource-constrained settings, Brazil’s tripartite collaboration between government, academia, and industry established a “tele-bariatric clinic” and mobile-health follow-up platform, completing 12,000 remote BS evaluations within two years and achieving a one-year follow-up rate of 78%, providing a replicable model for middle-income countries [28,29].

From a policy perspective, WHO’s 2022 “Accelerating Obesity Management Framework” urges nations to integrate obesity education into essential public-health functions via four pathways: (1) embed BS indications, benefits, and risks into mandatory CME credits for primary-care physicians; (2) develop evidence-based clinical pathways for “referral-assessment-surgery-follow-up” and monitor quality indicators in real time through health-information platforms; (3) reduce patient out-of-pocket costs through insurance reimbursement or dedicated funds and provide additional incentives to institutions that deliver standardized follow-up; and (4) leverage social media, community lectures, and peer-support groups to reduce obesity stigma and increase public acceptance of BS as chronic-disease therapy [37,38].

Returning to the present cross-sectional survey of Chinese community hospitals, we found that 79.4% of general practitioners had never received BS training, 85.2% had never participated in post-operative follow-up, 54.8% worked in institutions that offered no BS services, and 71.6% reported an absence of patient-education activities - figures that all significantly exceed comparable data from Europe and North America, underscoring an amplified version of the global barriers in China’s primary-care setting. Notably, only 24.5% of respondents agreed that “lifelong vitamin and trace-element supplementation is necessary,” and fewer than 30% were familiar with endoscopic metabolic therapies - clear gaps compared with current international guidelines. Univariate analysis also showed that physicians aged 31-40 years, with a master’s degree or higher, senior professional titles, and employment in tertiary grade-B hospitals scored significantly higher on knowledge indices, whereas “routine availability of BS in the hospital” was not significantly associated with referral willingness, confirming that “hardware access” does not automatically translate into “cognitive readiness” and highlighting the urgency of systematic training.

Nevertheless, this study has notable limitations: its cross-sectional design precludes causal inference; the sample is predominantly from Sichuan and Chongqing, limiting regional representativeness; and self-administered questionnaires are subject to social-desirability and recall biases. Future research should adopt multicenter, longitudinal cohorts, integrate objective knowledge assessments (e.g., OSCE stations) with real-world referral data, and evaluate the additive effects of structured CME and insurance incentives; patient perspectives should also be incorporated to explore the interaction of physician-patient cognitive gaps on surgical acceptance, thereby generating high-quality evidence for tailored obesity-integrated management models in China and globally.

In summary, the obesity epidemic has become a trans-regional, trans-economic, and trans-cultural global public health crisis. Although bariatric surgery is supported by robust evidence, its clinical uptake remains hindered by knowledge gaps and cultural stigma. Only through government-led, policy-education-payment synergistic reforms; evidence-based training and quality monitoring provided by academic institutions; and performance incentives and patient education at the hospital level can the full continuum of “identification-referral-surgery-long-term management” be realized in primary-care settings, ensuring that international evidence-based achievements are translated into tangible health benefits for the millions of patients living with obesity worldwide.

## Conclusions

This study reiterates the urgency of the global obesity crisis and underscores the pivotal role of bariatric surgery (BS) in curbing this epidemic. Robust evidence has consistently demonstrated the long-term efficacy of BS in the treatment of severe obesity and its associated metabolic disorders; nevertheless, real-world uptake remains markedly below expectations. The fundamental barrier is the widespread absence of systematic training and in-depth understanding among front-line healthcare professionals. In our community-hospital sample from China, more than 80% of general practitioners reported no prior BS training and 70% of institutions lacked patient-education programs, mirroring the compounded challenges - knowledge gaps, cultural reservations, and resource constraints - observed across Europe and North America. To reverse this trajectory, evidence-based BS content must be embedded as a mandatory component of primary-care continuing medical education, complemented by transnational knowledge exchange and remote-learning initiatives. Concurrently, reimbursement reform, performance-based incentives, and public destigmatization campaigns should be leveraged to establish an integrated governance framework that aligns government, academia, hospitals, and communities. Only through such coordinated efforts can BS be transformed from a highly specialized intervention into a universally accessible standard of care, thereby securing obesity management a definitive position on the global health-policy agenda.

## Figures and Tables

**Figure 3:**
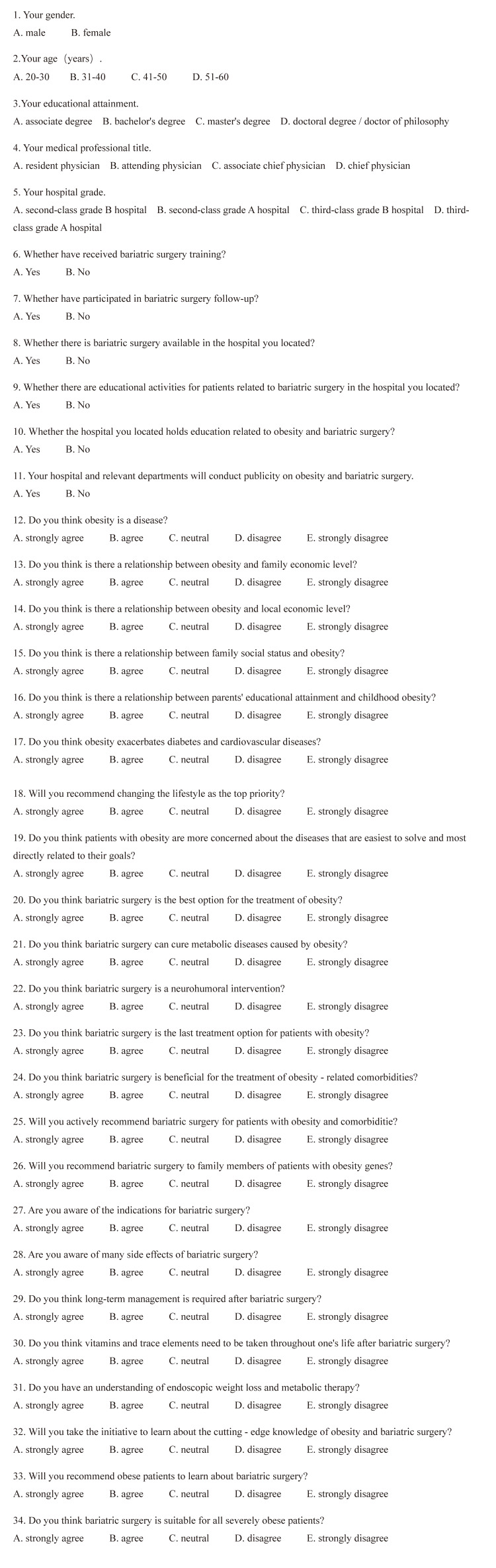
Electronic questionnaire

## Appendices

**Table 6:** STROBE Statement *Give information separately for cases and controls in case-control studies and, if applicable, for exposed and unexposed groups in cohort and cross-sectional studies. Note: An Explanation and Elaboration article discusses each checklist item and gives methodological background and published examples of transparent reporting. The STROBE checklist is best used in conjunction with this article (freely available on the websites of PLoS Medicine at http://www.plosmedicine.org/, Annals of Internal Medicine at http://www.annals.org/, and Epidemiology at http://www.epidem.com/). Information on the STROBE Initiative is available at www.strobe-statement.org.

	Item No	Recommendation
Title and abstract	1	①cross-sectional study；②Objective: The purpose of this study is to assess the awareness of primary care doctors in China about obesity and bariatric surgery. Methods: Between September and December 2024, an anonymous electronic questionnaire was distributed to doctors in primary care hospitals. The questionnaire included four sections: basic information, knowledge about obesity, understanding of weight loss surgery, and factors affecting the implementation of bariatric surgery, comprising a total of 47 questions. After the surveys were collected, data were compiled and analyzed according to participants’ gender, age, education, title, hospital, and department. Results: Among the respondents, 50.3% were male and 49.7% were female, with the majority being aged 31-40 years, and most holding a bachelor's degree. 79.4% of respondents had not received training in bariatric surgery, and 85.2% had not participated in postoperative follow-up. 54.8% of hospitals did not offer bariatric surgery, and 71.6% did not provide related educational activities. Most respondents believed obesity is a chronic disease related to diabetes and cardiovascular diseases, and recognized that obesity is associated with economic status and family social standing. Regarding bariatric surgery, 25.2% of respondents considered it the best treatment option, 40.7% recommended that family members consider the surgery, and 43.2% believed long-term management is necessary after the surgery. Conclusion: Primary care doctors in community hospitals have inadequate awareness of obesity and bariatric surgery, and most community hospitals do not prioritize obesity enough.
Introduction		
Background/rationale	2	The World Obesity Atlas 2024, published by the World Obesity Federation (WOF), projects that by 2035, 3.3 billion adults—equivalent to 54% of the global adult population—will be affected by overweight and obesity. According to the China Obesity Prevention and Treatment and Bariatric Surgery White Paper 2023, the average BMI and waist circumference of Chinese adults continue to increase, with the combined overweight and obesity rate exceeding 50%. Based on Chinese diagnostic criteria (BMI ≥ 28 kg/m²), China has the largest number of obese individuals globally. The obesity rate in China continues to rise, posing a serious threat to public health and constituting an urgent public health issue. Obesity affects nearly all organ systems and is associated with an increased risk of chronic diseases, disability, and premature mortality. Bariatric surgery, an effective and durable intervention for obesity and related metabolic disorders, has gained broad clinical acceptance and is now widely implemented in practice. Current research primarily focuses on patient acceptance and the long-term outcomes of bariatric surgery. However, the extent of clinicians’ understanding of obesity and bariatric surgery—despite their central role in healthcare decision-making—remains unclear. This study investigates Chinese primary care physicians’ awareness of bariatric surgery, aiming to improve medical guidance for obese patients and strengthen the scientific rigor and accuracy of clinical decision-making.
Objectives	3	The purpose of this study is to assess the awareness of primary care doctors in China about obesity and bariatric surgery.
Methods		
Study design	4	This study was a cross-sectional survey conducted between September and December 2024. A structured, self-designed electronic questionnaire was distributed anonymously to primary care physicians working in community hospitals across various regions of China. The questionnaire was developed based on the White Paper on Obesity Prevention and Bariatric Surgery in China (2023) and the WHO Obesity Management Guidelines (see Supplementary Material 1).
Setting	5	The data collection was carried out from September to December 2024 through online platforms. Questionnaire links were stratified and distributed according to hospital level and region to ensure representative sampling. The study was conducted in community hospitals across China, covering multiple provinces and hospital tiers.
Participants	6	(Inclusion criteria: Licensed general practitioners working in Chinese community hospitals. Voluntary participation with signed electronic informed consent. Exclusion criteria: Incomplete questionnaires (with a missing rate >10%). Physicians from non-clinical departments or not directly involved in patient care. A stratified random sampling approach was used, based on hospital level (private, secondary, tertiary) and geographical region. After excluding invalid responses, a total of 155 valid questionnaires were included in the analysis. Participants were categorized by: Age groups: 20–30, 31–40, 41–50, and 51–60 years. Educational level: associate degree, bachelor's, master's, doctorate. Job title: resident physician, attending physician, associate chief physician, chief physician. Hospital level: private hospitals, secondary A and below, tertiary B, and tertiary A hospitals.
Variables	7	The questionnaire was divided into four sections with 34 questions total: Basic Information (13 items): gender, age, education, title, region, hospital level, department, and whether the participant had received training in bariatric surgery. Knowledge of Obesity (9 items): understanding of obesity-related definitions, risk factors, and complications. Knowledge of Bariatric Surgery (11 items): awareness of indications, procedures, and effectiveness of surgical treatments. Perceived Barriers and Influencing Factors (4 items): factors that affect the adoption of bariatric surgery. Variables were analyzed across demographic groups to assess correlations with knowledge and attitudes toward obesity and bariatric surgery. The primary outcomes were physician awareness and understanding of obesity and bariatric surgery. Secondary variables included age, education, professional title, hospital level, and department affiliation. Potential confounding factors such as prior training in bariatric surgery were also considered.
Data sources/ measurement	8*	All data were obtained from self-administered electronic questionnaires completed by participating primary care physicians. Each question was designed with single or multiple-choice responses based on validated frameworks from the White Paper on Obesity Prevention and Bariatric Surgery in China (2023) and the WHO Obesity Management Guidelines. For knowledge-related sections, correct answers were predetermined based on guidelines. For attitudinal or perception-based questions, a Likert-type scale was used when applicable. All participants received the same version of the questionnaire, and responses were collected under identical conditions, ensuring methodological comparability across all subgroups.
Bias	9	To minimize selection bias, stratified random sampling was used across regions and hospital levels. Participation was anonymous and voluntary, reducing response bias. To control for information bias, questions were written in clear, standardized language, pilot-tested for comprehension by 10 general practitioners, and refined accordingly. Incomplete or ambiguous responses were excluded during data cleaning to reduce misclassification.
Study size	10	The minimum sample size was estimated based on a conservative expected awareness rate of 50% (to yield the largest required sample size), with a confidence level of 95% and a margin of error of 8%. Using the formula for estimating proportions in cross-sectional studies, the required sample size was calculated to be ~150 participants, after accounting for a 10–15% nonresponse rate. A total of 155 valid responses were ultimately collected.
Quantitative variables	11	Knowledge scores were calculated by assigning one point for each correct answer in the obesity and bariatric surgery sections. Total scores were used as continuous variables and also categorized into low, moderate, and high knowledge groups based on tertiles. Groupings for age, education, job title, and hospital level were pre-defined (see prior sections). These categorical groupings were used for subgroup comparison analyses.
Statistical methods	12	Descriptive statistics were used to summarize demographic variables and knowledge scores (mean ± SD, median, frequencies, and percentages as appropriate). Chi-square tests or Fisher’s exact tests were applied to compare categorical variables between groups. t-tests or ANOVA were used to compare continuous variables between subgroups. Multivariate logistic regression was employed to identify independent factors associated with high knowledge levels of bariatric surgery, controlling for confounders including age, education, and training experience. Missing data were minimal (<5%) and handled by listwise deletion. No imputation was performed. Sensitivity analyses were conducted by re-running key models excluding outliers and borderline responses. All statistical analyses were performed using SPSS version 26.0 (IBM Corp., Armonk, NY, USA). A two-tailed p-value < 0.05 was considered statistically significant.

Results		
Participants	13*	A total of 155 physicians were included in the final analysis. All participants completed the survey; there were no exclusions or dropouts. A flowchart was not included, as this was a cross-sectional survey with no follow-up period.
Descriptive data	14*	Participant characteristics such as gender, age, educational attainment, professional title, and hospital grade were reported (Table 1). The sample included 78 males (50.3%) and 77 females (49.7%), with the majority aged 31–40 years (49.1%). Most held a bachelor’s (55.1%) or master’s degree (40.3%). No missing data were reported for any demographic variables. As this was a cross-sectional study, follow-up time was not applicable.
Outcome data	15*	In this cross-sectional study, outcome data were reported as the proportion of participants who demonstrated awareness or attitudes regarding key topics such as bariatric surgery training, follow-up practices, institutional support, and perceptions of obesity and metabolic surgery. These outcomes were measured via self-reported Likert-scale responses and summarized using descriptive statistics. For example, 25.2% of physicians agreed that bariatric surgery is the best treatment option for obesity, and 43.2% acknowledged the need for long-term postoperative management.
Main results	16	Associations between physician characteristics and knowledge/attitudes were analyzed using chi-square tests and univariate analysis. Where applicable, estimates of association strength were reported using odds ratios (ORs) and 95% confidence intervals. For example: Physicians with bariatric surgery training were more likely to recommend surgery for patients with comorbidities (OR = 2.18, 95% CI: 1.31–3.62). Institutional patient education was associated with increased likelihood of surgery recommendation (OR = 2.91, 95% CI: 1.68–5.04). Confounding adjustment was limited to univariate comparisons due to the cross-sectional design. Variables such as age, education level, hospital grade, and training participation were compared across outcomes. No categorization thresholds were arbitrarily created for continuous variables, as most data were categorical or ordinal by design.
Other analyses	17	Subgroup analyses were performed by stratifying respondents based on: Training participation (trained vs. untrained), Institutional support (with vs. without patient education programs), Age group, and educational level. These stratified results helped identify groups with significantly higher levels of awareness or stronger attitudes toward bariatric surgery. No sensitivity analyses were conducted due to the descriptive and exploratory nature of the study.
Discussion		
Key results	18	This study found significant knowledge and attitude gaps among physicians regarding bariatric surgery. Despite strong international evidence supporting its efficacy, most participants reported limited familiarity with surgical indications and postoperative management. Physicians who received bariatric training or were in educationally supportive institutions were more likely to recommend surgery and understand long-term care needs.
Limitations	19	This study has several limitations. First, it was based on a cross-sectional survey and relied on self-reported data, which introduces potential recall and social desirability biases. Second, the sample was limited in size and geographically constrained, possibly affecting the generalizability of the findings. The lack of multivariate adjustment also restricts the interpretation of causal relationships. These limitations may underestimate or overestimate actual knowledge and behaviors.
Interpretation	20	The findings align with international literature that highlights underutilization of bariatric surgery despite clinical endorsement. Cultural stigma, inadequate provider training, and institutional barriers appear to influence physician behaviors across healthcare systems. This study reinforces the need for multi-level interventions, including CME programs and health policy reform. While the observed associations are consistent with prior studies, further longitudinal and multi-center investigations are needed to confirm causality and guide evidence-based policymaking.
Generalisability	21	Although the findings are most directly applicable to physicians in the sampled region, the identified trends—particularly around training gaps and inconsistent institutional support—mirror international reports. Therefore, the conclusions may be generalizable to similar urban healthcare systems, especially in other middle-income or rapidly developing countries. However, caution is advised when extrapolating to rural, resource-limited, or culturally distinct contexts.
Other information		
Funding	22	Doctoral Research Start-up Fund of North Sichuan Medical College (Grant No. CBY22-QDA15) Horizontal Project of the Affiliated Hospital of North Sichuan Medical College (Grant No. 2022LC005) Scientific Research Development Program of the Affiliated Hospital of North Sichuan Medical College (Grant No. 2024PTZK011) Self-funded Scientific Research Projects of the Affiliated Hospital of North Sichuan Medical College (Grant Nos. KYZC20250549, KYZX20250405, and KYZX20250534).
